# Phonon triggered rhombohedral lattice distortion in vanadium at high pressure

**DOI:** 10.1038/srep31887

**Published:** 2016-08-19

**Authors:** Daniele Antonangeli, Daniel L. Farber, Alexei Bosak, Chantel M. Aracne, David G. Ruddle, Michael Krisch

**Affiliations:** 1 Institut de Minéralogie, de Physique des Matériaux, et de Cosmochimie (IMPMC), UMR CNRS 7590, Sorbonne Universités - UPMC, Muséum National d’Hystoire Naturelle, IRD, 75252 Paris, France; 2 Department of Earth and Planetary Sciences, University of California Santa Cruz, Santa Cruz, California 95063, USA; 3 European Synchrotron Radiation Facility, 38000 Grenoble, France; 4 Lawrence Livermore National Laboratory, Livermore, California 94550, USA

## Abstract

In spite of the simple body-centered-cubic crystal structure, the elements of group V, vanadium, niobium and tantalum, show strong interactions between the electronic properties and lattice dynamics. Further, these interactions can be tuned by external parameters, such as pressure and temperature. We used inelastic x-ray scattering to probe the phonon dispersion of single-crystalline vanadium as a function of pressure to 45 GPa. Our measurements show an anomalous high-pressure behavior of the transverse acoustic mode along the (100) direction and a softening of the elastic modulus *C*_44_ that triggers a rhombohedral lattice distortion occurring between 34 and 39 GPa. Our results provide the missing experimental confirmation of the theoretically predicted shear instability arising from the progressive intra-band nesting of the Fermi surface with increasing pressure, a scenario common to all transition metals of group V.

Although body-centered-cubic (bcc) metals have one of the simplest crystal structures in the periodic table, they display a rich variety of physical properties and thus provide an important benchmark for the validation of modern first-principle theory^1^. In particular, the lattice dynamics of bcc transition metals have attracted great scientific attention. The Kohn anomaly in the phonon dispersion of bcc transition metals, and its dependence upon pressure and temperature, has been a challenge for first principle calculations to capture^2^,^3^. The strong differences displayed by the phonon dispersion of the various elements of group V (vanadium, niobium and tantalum) suggest that there is a profound dependence of the phonon energies on the electronic structure and the topology of the Fermi surface^4^,^5^. The high superconducting temperature (*T*_*c*_ = 9.25 K for Nb and *T*_*c*_ = 5.3 K for V) and its notable increase with pressure have also been suggested to be due to electron-phonon coupling and Fermi-surface properties^6^,^7^,^8^. The stability at high pressure of the bcc structure is speculated to critically hinge on the topology of the Fermi surface as well, and an intra-band nesting is theoretically predicted to give rise to shear phonon instabilities^9^.

Focusing on vanadium, calculations of shear instabilities arising from phonon softening^9^ have prompted the reinvestigation of the structural stability of V under high pressure. X-ray powder diffraction showed a transition from the bcc to a rhombohedral phase at 69 GPa^10^ and subsequent calculations have confirmed the nature of the rhombohedral distortion - even though different transition pressures were proposed^5^,^11^,^12^,^13^. Interestingly, under hydrostatic conditions the transition is hindered, and non-hydrostaticity helps in overcoming the energy barrier associated with the structural phase change^14^. Irrespective of the exact pressure at which the transition occurs, the bulk of theoretical work points towards a common mechanism: the progressive intra-band nesting at the Fermi surface that eventually leads to an electronic topological transition (ETT) with a concomitant transverse acoustic phonon mode softening. Specifically, at a critical pressure, parts of the 3rd electronic, partially occupied, conduction band of d symmetry move into the close vicinity of the Fermi level. The nesting vector, already responsible for the Kohn anomaly in the transverse acoustic phonon mode along the (*ξ*, 0, 0) direction at *ξ* = 0.25 at ambient pressure^8^, reduces to zero and the ETT takes place, with instability in the shear elastic constant *C*_44_^9^. This anomalous softening of the elastic response causes an energy gain that counterbalances the standard elastic strain energy cost, and ultimately leads to the rhombohedral lattice distortion. However, since experimental measurements across the transition were limited to x-ray diffraction^10^,^14^, a direct experimental verification of this theoretical picture was missing. Indeed, the phonon dispersion of vanadium has been difficult to measure as it is essentially an incoherent scatterer, which prevented the use of inelastic neutron scattering even at ambient conditions.

Here we present phonon dispersion measurements in vanadium at high pressure by inelastic x-ray scattering (IXS). This technique has been proven to be very suitable for the investigation of single crystal elasticity at high pressure^15^,^16^,^17^, and is capable of mapping phonon dispersion curves^3^,^18^,^19^. The fact that V is essentially an incoherent scatterer is not an actual limitation to the use of IXS^4^.

## Results and Discussion

**Figure 1:**
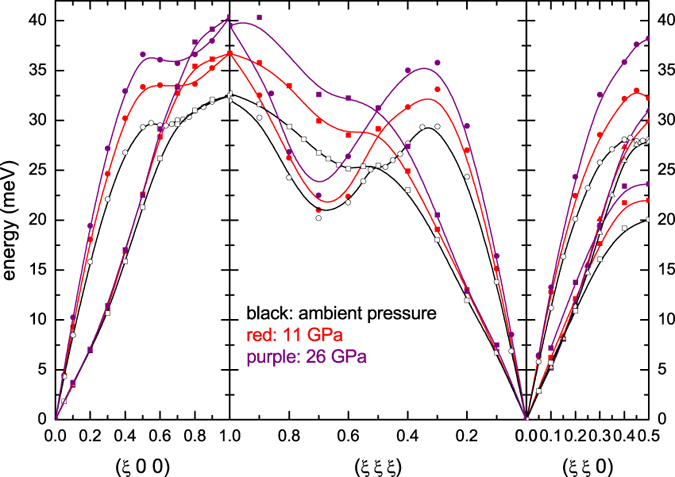
Phonon dispersion of vanadium along high symmetry directions, at ambient pressure (open black symbols^4^), 11 GPa (red symbols) and 26 GPa (purple symbols) at ambient temperature. Circles denote LA modes, squares and triangles (when not degenerate) denote TA modes. For clarity errors bars (between 0.1 and 1.2 meV) are not shown. The lines through the experimental points are guides to the eye.

**Figure 2:**
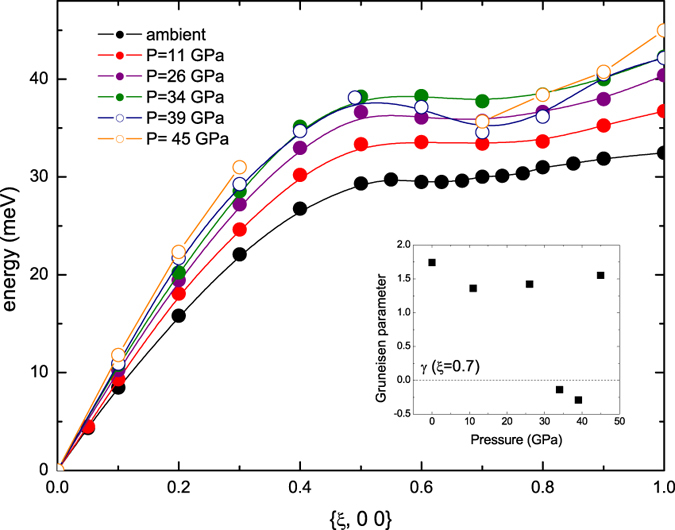
Pressure dependence of the LA dispersion along (*ξ*, 0, 0); bcc phase (solid circles), rhombohedral phase (open circles). Errors (between 0.1 and 1.2 meV) are smaller than the symbol size. The lines through the experimental points are guides to the eye. Dispersion at 45 GPa is incomplete. Inset: pressure evolution of the mode Gruneisen parameter (*γ*) for *ξ* = 0.7.

**Figure 3:**
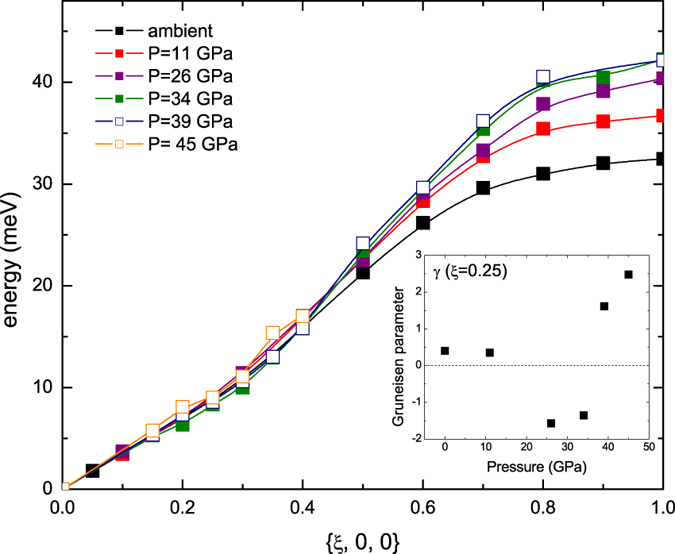
Pressure dependence of the TA dispersion along (*ξ*, 0, 0); bcc phase (solid squares), rhombohedral phase (open squares). Errors (between 0.1 and 1.2 meV) are smaller than the symbol size. The lines through the experimental points are guides to the eye. Dispersion at 45 GPa is incomplete. Inset: pressure evolution of the mode Gruneisen parameter (*γ*) for *ξ* = 0.25.

At 11 and 26 GPa we measured the complete phonon dispersions for the high symmetry directions, which are displayed in Fig. 1 together with the dispersion at ambient conditions^4^. A qualitative comparison indicates that dispersions along the (*ξ*, *ξ*, 0) and (*ξ*, *ξ*, *ξ*) directions retain the same overall shape as at ambient conditions, but are shifted to higher energies as commonly expected upon compression. Noteworthy, the behavior along (*ξ*, 0, 0) is more intriguing. Indeed, while the longitudinal acoustic (LA) mode stiffens, the transverse acoustic (TA) mode does not exhibit any significant energy shift below *ξ* = 0.3, and only changes for high q values. Therefore, at higher pressure, we focused on the behavior of the LA and TA modes along the (*ξ*, 0, 0) direction. Considering the low-q part of the dispersions, we notice a phonon stiffening for the LA mode, as commonly expected with increasing pressure (Fig. 2), while there is no significant change in the TA mode, except for a slight but still visible softening at *ξ* between 0.2 and 0.3, up to 34 GPa (Fig. 3). At higher pressure the TA mode gets stiffer, and the anomaly seems less pronounced. This effect is highlighted in the inset of Fig. 3, which shows the mode Gruneisen parameter (*γ*) for *ξ* = 0.25 as a function of pressure. Upon initial pressure increase, the phonon energy at *ξ* = 0.25 does not significantly vary (*γ* is close to zero), and then starts to soften, as evidenced by the negative *γ* values. At higher pressures (39 and 45 GPa), the commonly expected positive *γ* values are recovered. A similar trend is observed for *ξ* = 0.2 and *ξ* = 0.3 as well. Qualitatively, this behavior was predicted by ab initio calculations^2^, even though there are not-negligible, quantitative differences in the pressure range and the magnitude of the effect. In particular, the measurements cannot confirm the expected progressive shifting of the Khon anomaly to smaller q value^9^. An analysis of the overall shape of the LA dispersion highlights a change above 34 GPa, with a local minimum for *ξ*~0.7 visible at 39 and 45 GPa, suggesting a distinct electronic feature of the tetragonal phase. Once again, this effect is highlighted by the negative *γ* values for *ξ* = 0.7 at 34 and 39 GPa (inset of Fig. 2).

**Figure 4:**
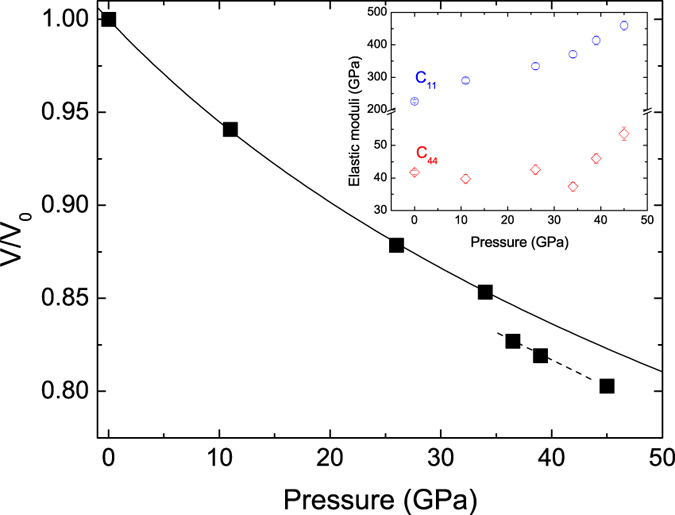
Compression curve of vanadium up to 45 GPa. The black squares represent the volume estimated from (110) and (002) reflections of our single-crystal (errors are smaller than the symbol size). The solid line is the equation of state for the bcc phase from literature^10^. The dashed line is a guide to the eye. The inset shows the elastic moduli *C*_11_ and *C*_44_ as a function of pressure.

Importantly, the enhancement of the Kohn anomaly with pressure is predicted to occur with an associate softening of the shear elastic modulus *C*_44_^9^. From the initial slope of the phonon dispersion of the longitudinal acoustic and transverse acoustic modes along the (*ξ*, 0, 0) direction, we have derived the elastic moduli *C*_11_ and *C*_44_ by solving the Christoffel equation^20^. We report these as a function of pressure in the inset of Fig. 4. While we note a monotonic increase in *C*_11_, *C*_44_ remains essentially constant up to 26 GPa, softens at 34 GPa, and then increases towards even higher pressures. This anomalous behavior is overall consistent with the theoretical predictions^5^,^9^,^11^,^12^,^13^ for *C*_44_ at pressures approaching the electronic topological transition. Still, a few differences remain between the experiments and the calculations: the magnitude of the softening is more pronounced in the calculations and the measurements suggest an absence of a significant hardening upon initial compression. Nonetheless, our data are the first direct measurements of the mechanism responsible for the rhombohedral lattice distortion, i.e. the softening of *C*_44_. Our conclusions seem to be further supported by x-ray diffraction data collected on V single crystals in parallel with the IXS measurements. The estimated volumes well follow the equation of state for the bcc phase from literature^10^ up to 34 GPa, whereas a departure is observed starting from 36 GPa (Fig. 4). Such a volume discontinuity (~2%) was not reported in previous studies where measurements were carried out on powders^10^,^14^. This support the first-order nature of the phase transition, in agreement with a group theory analysis that indicates that the zone-centre transition from Im3m to R-3m has necessarily to be of first order. The first-order nature of the transition was as well advocated by a recent study within the frame of the Landau theory^21^. Further, more detailed diffraction measurements on single crystals are needed to clarify this point.

Our work places the onset of the rhombohedral distortion between 34 and 39 GPa, in disagreement with original x-ray diffraction measurements^10^, but in quite good agreement with the most recent diffraction work and with the hypothesis that the true thermodynamic value of the transition pressure is close to 30 GPa^14^. As already mentioned, under purely hydrostatic compression, the phase transition is hindered. Our measurements have been performed on relatively big single crystals, which are much more sensitive to non-hydrostaticity than powders. In particular, the crystals were cut with the (110) direction normal to the surface and loaded in the DAC with the (110) direction along the compression axis of the cell. Even when noble gases are used as pressure transmitting medium, a small deviatoric stress component along the main compression axis of the cell is a common feature (at room temperature Ne solidifies around 5 GPa and He around 12 GPa, so none of the media is truly hydrostatic). Thus, we argue that such a small non-hydrostatic component along the (110) direction allowed overcoming the energy associated to the phase change^14^.

## Conclusions

We report phonon dispersion measurements in vanadium at high pressure. Our experimental results provide a coherent picture of the nature of the high-pressure behavior of group V transition metals, which critically hinges on the interplay between the lattice dynamics and the electronic structure at the Fermi energy. For V, Nb and Ta, increasing pressure promotes the shift of the s-derived electronic band with respect to the d-derived bands and, at a critical pressure, the distorted octahedron hole-pocket around the Γ point shrinks and the connected opened hole-tube terminates at the (*ξ*, 0, 0) direction^9^. Such specific features of the Fermi surface cause a reduction of the nesting vector and we argue that this leads to the softening of *C*_44_ we document here. The consequent shear distortion lifts degeneracies of electron bands close to the Fermi energy, leading to an energy gain as a function of the distortion. This energy gain at least partially counterbalances the elastic strain energy cost, resulting in an anomalous softening of the elastic response. Upon further pressure increase, the relevant features in the electronic bands move away from the Fermi level, and the system recovers its normal behavior. We stress, however, that in spite of the similarity in the topology of the Fermi surface among all the transition metals in group V, the progressive intra-band nesting of the Fermi surface and the associated transverse acoustic phonon mode softening reduces moving from period 4 (V) to period 6 (Ta). V is the only case in which the energy gain is large enough to lead to sizable distortion and to a structural phase transition. Substitution of V with Nb, even to a fraction as small as 5 at.% removes the lattice instability^9^, while retaining the softening of *C*_44_. For Ta the shear softening occurs at much higher pressure and is smaller^22^, and the bcc structure is maintained.

## Methods

High quality vanadium single crystals, with direction normal to the sample surface oriented along the (110) direction, were prepared by fs laser cutting, with subsequent chemical etching^23^. Samples of ~30 *μm* diameter and ~15 *μm* thickness were loaded in membrane-driven diamond anvil cells (DAC) equipped with 300 or 350 *μm* culets diamond and rhenium gaskets. To ensure quasi-hydrostatic compression, helium or neon was used as pressure transmitting medium. As in our previous experiments^15^, we did not notice any appreciable difference between data collected using helium or neon, suggesting that possible He diffusion within the samples is likely negligible (or at least that both He and Ne have similar effects).

IXS measurements were carried out on beamline ID28 at the European Synchrotron Radiation Facility, using the Si(9, 9, 9) instrument configuration, which provides an overall energy resolution of 3 meV full-width-half-maximum (FWHM). The direction and size of the momentum transfer were selected by an appropriate choice of the scattering angle and the sample orientation in the horizontal scattering plane. The momentum resolution was set to 0.28 *nm*^−1^ and 0.84 *nm*^−1^ in the horizontal and vertical planes, respectively. The focused x-ray beam of 30 × 90 *μm*^2^ FWHM was further reduced in the vertical direction down to 40 *μm* by slits in order to more closely match the sample dimensions. Measurements have been performed in transmission geometry, with the incoming x-ray beam impinging along the cell axis, through the diamonds. We collected data at room temperature and pressures of 11, 26 and 34 GPa (cubic phase), 39 and 45 GPa (tetragonal phase) as determined from ruby fluorescence measurements. Examples of IXS spectra are shown in Supplementary Figure S1 (online). The energy position E(q) of the phonons was extracted by fitting the inelastic contribution by a Lorentzian function convoluted with the experimental determined resolution function, utilizing a standard *χ*^2^ minimization routine.

Orientation of the crystals on the spectrometer was accomplished using an in-line CCD camera. This allowed us to place the appropriate Bragg reflection in the horizontal plane of the spectrometer as well as defining the orientation matrix. By scanning the scattering angle at the elastic energy (i.e. q-scan at Δ*E* = 0) we collected (110) and (002) reflections, directly deriving sample density, monitoring the crystal quality via rocking curve width analysis (online Supplementary Figure S2). At 45 GPa we measured the (112) reflection as well. Rocking width of the (110) reflection in the bcc phase was consistently around 0.2 degree, independently of the actual pressure. Broadening of the (110) reflection collected at 36 and 39 GPa and of the (110) and (112) reflections collected at 45 GPa were interpreted as signature of the rhombohedral distortion^14^ (see Supplementary Figure S3).

Further details of the experimental setup and data analysis can be found elsewhere^3^,^15^,^24^.

## Additional Information

**How to cite this article**: Antonangeli, D. *et al*. Phonon triggered rhombohedral lattice distortion in vanadium at high pressure. *Sci. Rep.* **6**, 31887; doi: 10.1038/srep31887 (2016).

## Supplementary Material

Supplementary Information
