# Supplementary material for: Phonon triggered rhombohedral lattice distortion in vanadium at high pressure
Source: Sci Rep. 2016 Aug 19;6:31887. doi: 10.1038/srep31887 (PMC4990969; doi:10.1038/srep31887)
Supplement: Supplementary Information [file srep31887-s1.pdf]

# Supplementary Information for

## Phonon triggered rhombohedral lattice distortion in vanadium at high pressure

Daniele Antonangeli[1,2,*], Daniel L. Farber[3,2], Alexei Bosak[4], Chantel M. Aracne[2], David G. Ruddle[2], and Michael Krisch[4]

[1]Institut de Minéralogie, de Physique des Matériaux , et de Cosmochimie (IMPMC), UMR CNRS 7590, Sorbonne Universités - UPMC, Muséum National d'Hystoire Naturelle, IRD, 75252 Paris, France

[2]Lawrence Livermore National Laboratory, Livermore, California 94550, USA

[3]Department of Earth and Planetary Sciences, University of California Santa Cruz, Santa Cruz, California 95063, USA

[4]European Synchrotron Radiation Facility, F-38043 Grenoble, France

* daniele.antonangeli@impmc.upmc.fr

**This PDF file includes:**

Supplementary Figure S1

Supplementary Figure S2

Supplementary Figure S3

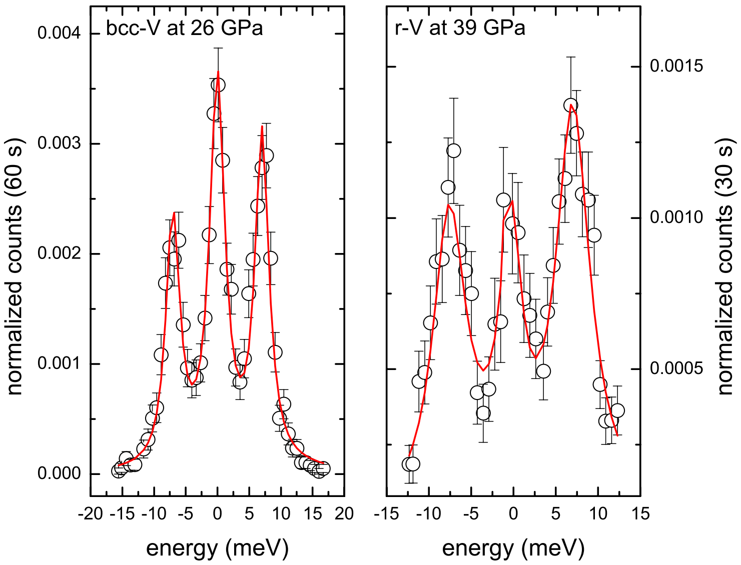


**Figure S1**. Representative IXS spectra of bcc (left) and rhombohedral (right) vanadium at high pressure. The experimental spectra of the TA phonon along the (ξ, 0, 0) direction for ξ=0.2 (open circles) are shown together with their best fits (red lines).

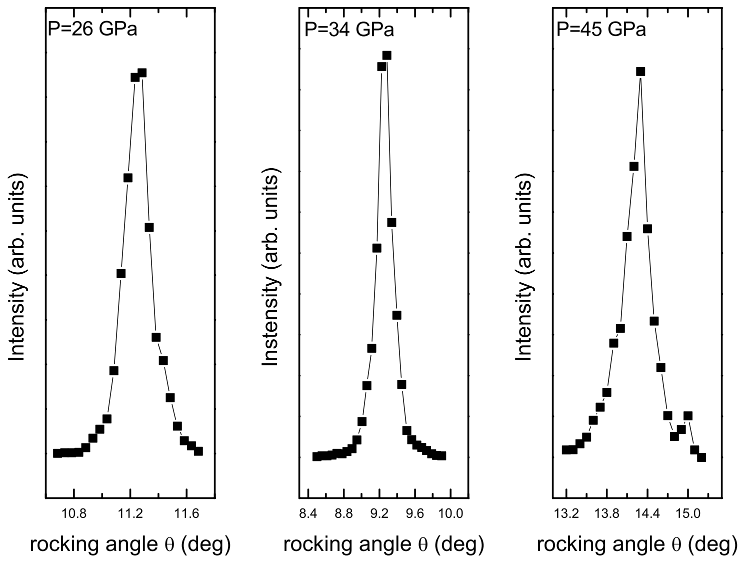


**Figure S2**. Representative rocking curves of the (110) reflection of bcc (left and center) and rhombohedral (right) vanadium at high pressure.

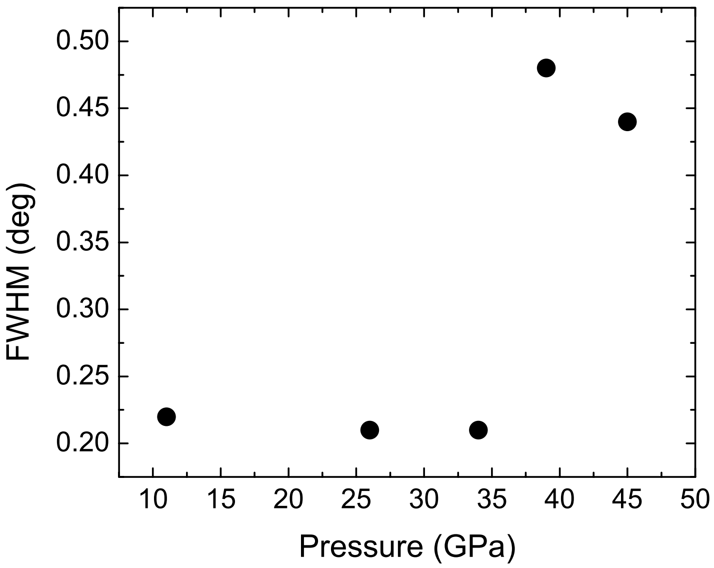


**Figure S3**. Rocking width (full width half maximum) of the (110) reflection as a function of pressure. Errors are smaller than the symbol size. Broadening of the (110) reflection at 39 and at 45 GPa is interpreted as signature of the rhombohedral distortion.
